# Editorial: Advances in ME/CFS Research and Clinical Care

**DOI:** 10.3389/fped.2019.00370

**Published:** 2019-09-18

**Authors:** Kenneth J. Friedman, Lucinda Bateman, Alison Bested, Zaher Nahle

**Affiliations:** ^1^Retired, Plantation, FL, United States; ^2^Bateman Horne Center, Salt Lake City, UT, United States; ^3^Integrative Medicine, Nova Southeastern University College of Osteopathic Medicine, Fort Lauderdale, FL, United States; ^4^Arthritis National Research Foundation, Long Beach, CA, United States

**Keywords:** Myalgic Encephalomyelitis, Chronic Fatigue Syndrome, Myalgic Encephalomyelitis/Chronic Fatigue Syndrome, ME/CFS, chronic illness, stigmatized illness, diagnosis, treatment

*Advances in ME/CFS Research and Clinical Care* spotlights Myalgic Encephalomyelitis/Chronic Fatigue Syndrome (ME/CFS): a maligned, stigmatized, under-researched disease, which lacks a definitive, objective clinical test for its diagnosis, and definitive palliative and curative treatments. A few brave physicians attempt to alleviate the suffering of the afflicted. They rely upon the patients' symptoms to guide them. Physicians can provide symptomatic relief and improve upon patients' abnormal physiological and metabolic parameters by intervening to cause the latter to approach normal limits. Documented to be more severely disabling than HIV-AIDS, ME/CFS receives disturbingly little funding in the United States and around the world. ME/CFS patients constitute an identifiable, underserved population that is in need of the recognition which would raise them from their current, underserved or non-served patient status into the mainstream of healthcare worldwide. ME/CFS is a common disease worldwide, affecting approximately 1 percent of the world's population.

Despite these obstacles, and as evidenced by the articles contained herein, ME/CFS research **is** being conducted, and patient care issues **are** being addressed. Today, researchers and clinicians communicate rapidly via the internet to overcome conventional impediments to knowledge and patient care.

At the end of the twentieth and the beginning of the twenty-first century, it seemed that the United States government had finally taken the lead in promoting research and patient care for a disease which had been described in exquisite detail by its own Public Health Service in the 1930's and subsequently largely ignored, or worse, defamed. More modern efforts to inform the U.S. Department of Health and Human Services (DHHS) began with the Chronic Fatigue Syndrome Coordinating Committee from 1996 to 2001, followed by reorganization as the Chronic Fatigue Syndrome Advisory Committee (CFSAC). That committee advised the U.S. Secretary of Health and Human Services on matters related to ME/CFS, but the recommendations of the CFSAC were largely ignored until 2015. That is when the Institute of Medicine (IOM) completed an evidence-based review and published a report, commissioned in response to a recommendation from the CFSAC, and sponsored by funds from the Office of Women's Health within DHHS, the National Institutes of Health (NIH), the Centers for Disease Control (CDC), the Food and Drug Administration (FDA), the Agency for Healthcare Research and Quality (AHRQ), and the Social Security Administration (SSA). The charge to the IOM committee was to develop clinical diagnostic criteria for ME/CFS, based on the evidence, and with the input of ME/CFS stakeholders. That report described a serious health crisis, an illness characterized by significant impairment and disability, inadequate diagnostic tools, barriers to healthcare access and trained physicians, high economic costs, and lack of treatment guidelines. The report contained a dissemination plan for education of U.S. medical institutions. In the 2 years that followed, the CFSAC systematically made such recommendations to the U.S. government agencies, in terms of both research support and patient care, which may have contributed to the demise of the CFSAC. In September of 2018, the Department of Health and Human Services decided not to renew the charter of the CFSAC.

It is promising that oversight of ME/CFS research has been moved from the Office of Women's Health to the National Institute of Neurological Diseases and Stroke (NINDS), and that a Trans-NIH Working Group, with members from several NIH Institutes, has been reinvigorated. The NIH is conducting a small but comprehensive inpatient study of early, post-viral ME/CFS, and has funded three Collaborative Research Centers and a central data management center.

Unfortunately, there is currently no leadership group in the U.S. government tasked with promoting ME/CFS patient care or provider education.

*Advances in ME/CFS Research and Clinical Care* makes the statement that despite these impediments, the compassion of the human spirit embedded in researcher, clinician, and caregiver boldly steps into this void, doing what is necessary to advance the science of, and treatment for people with, ME/CFS. Some members of the research and medicine communities have joined us to accelerate these goals. We welcome additional partners.

Our monograph starts with Friedman, “Advances in ME/CFS—Past, Present and Future,” which provides a brief history of the struggle for recognition of ME/CFS as a disease, and the struggles to establish ME/CFS research and clinical care.

Since patients do not exhibit an easily identified biomarker, abnormal metabolic or pathophysiological finding, ME/CFS is diagnosed largely by patient reported symptoms. Consequently, identifying the cause, the trigger, or triggers of ME/CFS is an ongoing field of investigation. This issue provides three contributions to that discussion and the literature: (1) Chu et al. look at patterns of ME/CFS onset and attempt to correlate it with the course of the disease, (2) Perez et al. discuss the possibility of genetic predispositions for immune system, hormonal, and metabolic dysfunctions as contributory triggers of ME/CFS, and (3) Kerr provides evidence for Epstein-Barr-virus induced gene upregulation being disease inducing in a subset of patients.

ME/CFS is a multi-organ system disease with high variability among patients. One patient's most severe symptoms or most affected organ systems differ from those of another. Thus, the questions arise: What symptoms best characterize the disease? What symptoms are mandatory to diagnose ME/CFS? How can we make diagnosis as easy as possible for the clinician? These questions lie within the domain of ME/CFS case definition. This issue contains two papers relevant to case definition. Jason and Sunnquist give some idea of the complexities involved when considering case definitions. The importance of an accurate diagnosis is considered by Geraghty and Adeniji.

Without standardized methodology for validating a ME/CFS diagnosis, researchers are searching for indirect methodologies, as evidenced by three papers in this issue: (1) Nacul et al. propose hand grip strength as a, “clinical biomarker,” of ME/CFS and also as an index of disease severity, (2) Stevens et al. discuss the use of 2-day, cardiopulmonary exercise testing to assess exertion intolerance in ME/CFS patients, and (3) Van Campen et al. discuss the lack of sensitivity of abbreviated tilt table testing for diagnosing postural tachycardia syndrome in ME/CFS patients—a common symptom found in ME/CFS.

A consequence of no standardized methodology for validating a diagnosis of ME/CFS is the difficulty in determining the number of individuals within a given population who suffer from the disease. In the United States, up to this time, only sampling techniques have been used to estimate prevalence. We are, therefore, pleased to present here a second methodology: Valdez et al. estimate the prevalence of ME/CFS by utilizing a large, medical claims database of a commercial insurance provider which they further analyzed using machine learning. Their approach yields data not only on current provider diagnosis of CFS and ME, gender, demographics and costs, but on estimated prevalence which is at variance with the random sampling data exclusively used previously. Obtaining different estimates by use of different methodologies suggests that additional studies need to be completed before the question of prevalence and other important questions can be answered with confidence.

We provide three papers representing the range of current, ongoing ME/CFS laboratory research: (1) as with other diseases, the microbiome is now being implicated in ME/CFS. Proal and Marshall put forward evidence that gastrointestinal pathogens are able to interfere with a patient's metabolism, gene expression, and immunity, (2) VanElzakker et al. contribute a critical review of the literature discussing the involvement of neuroinflammation and cytokines in ME/CFS, and (3) Lacerda et al. describe a UK ME/CFS Biobank, providing opportunity for new and further exploration of tissue abnormalities in ME/CFS.

We also provide three papers relevant to clinical ME/CFS research. Two of these papers, Van Campen et al. and Davenport et al. concern the cardiovascular symptoms of ME/CFS. The third, Boneva et al. indicates how a common co-morbidity of ME/CFS can influence the symptoms of the disease.

Regardless of the lack of knowledge of the etiology and pathology of ME/CFS, all patients are entitled to good healthcare. Clearly, providing healthcare for patients with a disease of unknown etiology, and highly variable, and waxing and waning symptoms, is a healthcare-provider challenge. Our monograph provides a number of articles to assist in that process: Lapp provides guidance for primary care physicians in dealing with the unique and challenging aspects of initially diagnosing and managing patients with ME/CFS. However, as Bae and Lin document, appropriate healthcare eludes many ME/CFS patients. One reason, in the United States, is the difficulty patients experience in qualifying for healthcare insurance benefits. Comerford and Podell provide guidance for medical providers on documenting the disabilities of the ME/CFS patient.

While the principles of medical treatment apply to all ME/CFS patients, pediatric, and adolescent patients have additional needs. We provide 4 articles describing the unique aspects of providing care to pediatric and adolescent patients. To start, Roma et al. describe the impact of core symptoms on the quality of life of a North American population of adolescents and young adults with ME/CFS. Knight et al. describe school functioning in adolescents with ME/CFS. Newton describes the challenges young people with ME/CFS face in the school environment, how these challenges can be overcome, and the role of the treating physician in this process. Finally, Rowe provides a retrospective view of what patients with ME/CFS felt benefitted them the most when in their adolescent, school-age years.

This monograph, despite its excellent and informative articles, lacks any article focused on what is termed the severely affected: those patients so afflicted by ME/CFS that they are unable to leave their homes or rise up out of their beds. This silent cohort of ME/CFS patients, estimated to be as high as 25 percent of the ME/CFS population, has never appeared in the peer-reviewed ME/CFS literature. The interest in the articles contained herein has given rise to the invitation to create a subsequent, invited, themed issue, entitled, “ME/CFS—The Severely Affected.” Clinicians and researchers are writing articles for that issue now. When completed, a description of ME/CFS throughout the range of its severity, and the resources that can be martialed to treat patients suffering from ME/CFS, will finally be available in the medical literature.

## Author Contributions

All authors listed have made a substantial, direct and intellectual contribution to the work, and approved it for publication.

### Conflict of Interest Statement

The authors declare that the research was conducted in the absence of any commercial or financial relationships that could be construed as a potential conflict of interest.

